# The genetic origins of species boundaries at subtropical and temperate ecoregions in the North American racers (*Coluber constrictor*)

**DOI:** 10.1038/s41437-024-00737-7

**Published:** 2024-11-28

**Authors:** Frank T. Burbrink, Edward A. Myers

**Affiliations:** 1 Department of Herpetology, American Museum of Natural History, New York, NY USA; 2 Department of Herpetology, California Academy of Sciences, San Francisco, CA USA

**Keywords:** Adaptive radiation, Ecology

## Abstract

Phylogeographically structured lineages are a common outcome of range-wide population genetic studies. In the southeastern United States, disconnection between populations found at the intersection of the southeastern coastal plains of peninsular Florida and the southeastern plains of the adjacent continent is readily apparent among many plants and animals. However, the timing and maintenance of species boundaries between these distinctly different subtropical and temperate regions remains unknown for all organisms studied there. Using genome-scale data, we examine the timing of origins, gene flow, and the movement of genes under selection in unique ecoregions within the North American racers (*Coluber constrictor*). Isolation-migration models along with tests of genome-wide selection, locus-environment associations, and spatial and genomic clines demonstrate that two unrecognized species are present and are in contact at the boundary of these two ecoregions. We show that selection at several loci associated with unique environments have maintained species boundaries despite constant levels of gene flow between these lineages over thousands of generations. This research provides a new avenue of research to examine speciation processes in poorly studied biodiversity hotspots.

## Introduction

The discovery of geographically structured phylogeographic lineages is common for most groups of plants and animals with appreciably large range sizes (Avise 2000; Soltis et al. 2006; Kumar and Kumar 2018). Where this genetic-geographic structure occurs, the timing of origin, changes in population size over time, and degree of genomic disconnection often relate to differences in the environment between lineages, changes in suitable habitat through time, and geographic mode of divergence (Manel et al. 2003; Wood et al. 2008; Schield et al. 2017; Burbrink and Ruane 2021). Understanding the demographic history of a species complex highlights the role that environment plays in isolating lineages (Wang and Bradburd 2014; Provost et al. 2021; Jaynes et al. 2022). This also helps better define the effect of biogeographic barriers on communities when studied in aggregate (Leaché et al. 2020; Edwards et al. 2022). How these lineages are maintained and thus discoverable over long periods of time, however, is less well known.

Upon formation of lineages, here defined as either populations or species, gene flow can exist either via constant connection through time, through secondary contact, or multiple waves of connection and disconnection (He et al. 2019). It is expected that extensive genome-wide hybridization even over short periods of time should collapse lineages (Taylor et al. 2006; Vonlanthen et al. 2012). However, for many taxa, persistent gene-flow has not disrupted the integrity of geographic lineages, even when inferred hybrid zones are large (Nadeau et al. 2012; Irwin et al. 2018; Burbrink et al. 2022). This suggests that gene flow is not constant across the entire genome and that alleles from particular loci do not successfully migrate between parental lineages. Indeed, studies of islands of genomic divergence have underscored that migrational heterogeneity is common (Nosil 2012; Wolf and Ellegren 2017). However, identifying these genomic islands and loci that do not freely cross hybrid zones is only now becoming possible with large scale genomic datasets (Hejase et al. 2020).

One area where lineage formation and speciation are common is at the connection between the Florida peninsula and the continental United States. This area was previously disconnected during the low glacial and higher sea level periods during the early Pleistocene, when central Florida existed as an archipelago (Webb 1990; Lane 1994). Additionally, peninsular Florida has a distinct subtropical climate, the southeastern coastal plains, compared to the continental Nearctic, southeastern plains (Bailey 1995). It is likely that allopatric isolation during the early Pleistocene or local adaptation to contrasting continental and subtropical environments resulted in lineage formation in numerous plants and animals (James 1961; Soltis et al. 2006; Fontanella et al. 2008; Burbrink and Guiher 2015; Marsico et al. 2015; Weinell and Austin 2017; Fetter and Weakley 2019; Lyman and Edwards 2022; Jones et al. 2023). Interestingly, this region is considered a biodiversity hotspot (Noss et al. 2015) yet there are few studies examining the origins of species and buildup of biodiversity in this region. While numerous phylogeographic lineages have been discovered here, with divergence times ranging from throughout the Pleistocene to the Miocene, no study has examined the connection between continental and Florida lineages and, specifically, how species have remained distinct given continuous geographic distributions and gene flow between lineages.

Previous studies have demonstrated that the North American racers (*Coluber constrictor*) show a major phylogeographic disconnection between Florida and the continent (Burbrink et al. 2008; Myers et al. 2017, 2024). Deep mtDNA divergence suggests this disconnection is ancient (~6 mya), however recently it was indicated using genome-scale data that divergence was younger with no gene flow between lineages. Here we explore, given the deep structure in both mtDNA and genomic datasets, how these interacting lineages have remained unique. We examine genome and spatial clines of divergence across all loci and environmental selection on loci throughout the genome. This study provides insight on how interacting lineages at the Florida peninsula have remained distinct over many thousands of generations despite hybridization.

## Methods

### Data

Using a target capture approach, we sequenced the squamate conserved loci (SqCL; Singhal et al. 2017) from 91 individuals across the range of North American racers. For details on DNA extraction, probe set, sequencing, raw sequence processing, and quality control regarding missing data for individuals and loci see Myers et al. (2024). For all analyses, we used only the 67 individuals previously found to be in the East and FL lineages (Myers et al. 2024). These samples also target the area where both lineages meet in north Florida. These data are available from the NCBI SRA under BioProject ID:PRJNA1082780 and from Dryad (doi: 10.5061/dryad.rxwdbrvhh).

### Population structure

We used both Discriminant Analysis of Principal Components (DAPC; Jombart et al. 2010) and *TESS3r* (Caye et al. 2016) to examine population structure at the FL-continental boundary. With the *adegenet* package (Jombart 2008) in R (R Core Team 2010) we transformed data using principal component analysis (PCA) with 150 axes and maximum of 20 groups. We retained all discriminant axes with eigenvalues > 1% and reduced the probability of PCA generating arbitrary groupings by taking the difference between actual and randomized cluster assignments and then calculating the optimal number of axes out of 150 to eliminate bias. We then used DAPC, which applies K-means to sequentially estimate the minimum number of genetic clusters and assign individuals into those clusters without prior group identification. Given the optimal number of axes, we ran DAPC and chose the number of groups with the lowest BIC (Bayesian information criterion) and AIC (Akaike information Criterion) values. Groups were cross validated using 90% training and 10% test datasets and the average predicted success for each group was estimated. The identity of loci with contributions to the first discriminant function greater than 99% of all loci were saved and used for downstream comparison of tests examining selection across the genome.

We also used the *TESS3r* package in R (Caye et al. 2016) to produce ancestry coefficients while accounting for geography using the graph-based nonnegative matrix factorization algorithm. Geographic coordinates were used for all samples to account for geography. We estimated K = 1–10 groups using 200 iterations and predicted where increasing values of K yielded diminishing values of root mean squared error (RMSE). Importantly, this method always produces lower RMSE values with increasing numbers of K, however, a threshold where K-values fail to generate geographically meaningful groups was applied (Burbrink et al. 2021).

We also examined the direct effect of space on genetic structure across all samples and within groups. Mantel tests have been shown to perform poorly using distance data to examine spatial structures (Legendre et al. 2015). We therefore accounted for the effect of geographic distances on genetic structure by transforming spatial distance among all samples using principal coordinates neighbor matrices (PCNM) in the R package *vegan* (Dixon 2003). This has the benefit of accounting for multiple spatial structures (neighborhoods) on genetic structure (Borcard and Legendre 2002). We calculated these relationships using redundancy analysis (RDA) and tested for significance using a Constrained Correspondence Analysis (CCA) ANOVA. We then determined the significance for each spatial predictor axis (Legendre et al. 2011) on genetic structure (McGaughran et al. 2014).

For the two lineages inferred here and in Myers et al. (2024), FL and East lineages, we estimated the fixation index (Fst) using the function pairwise. WCFst() in the R package *hierfstat* (Goudet 2005). This value and associated significance is used to confirm results from *TESS3r* and DAPC.

### Environmental-genomic relationship

To specifically examine the differences between the East and FL lineages in the context of alleles, space, and environment, we used we the unsupervised machine-learning, self-organizing maps (SOM or “Kohnen”) method described generally in Oja and Kaski (1999), Wehrens and Buydens (2007), and Wehrens and Kruisselbrink (2018) and specifically for population clustering in Pyron (2023). The SOMs are artificial neural networks that use competitive learning and can be extended to include multiple layers (sets of genetic, space, and environmental input data). Here, each layer has a unique influence on the two-dimensional output grid using estimated weights from each layer. The SOMs applied here yielded a two-dimensional representation of higher-dimension input layer space, ultimately representing clusters of individuals with similar values across these variables. The number of clusters (K) were determined by using K-means clustering of occupied cells into proximate units based on the weighted sum of squares of neighbor distances between adjacent cells. Using the R package *delim-som* (https://github.com/rpyron/delim-SOM) we used the 67 East and FL individuals, removing loci with missing data > 20%, yielding a total of 2964 SNPs (from 3143 SNPs). We also used georeferenced data for each individual to account for space and used the following environmental variables: Bioclim 1–19, elevation, net primary productivity (mean monthly MODIS Normalized Difference Vegetation Index; NDVI), and percentage of woody plants (datasets downloaded from http://www.paleoclim.org/ and https://github.com/rebeccalpowell/grassmapr). These variables were extracted for each genetic sample location using *raster* (Hijmans et al. 2014) in R. We also removed correlated environmental variables (ρ > 90%). We then made the 6 × 6 hexagonal SOM grid, used 100 repeats and 100 steps in the *Climate.SOM()* function on alleles, space, and environmental data to generate numbers of input layer weights, clusters, neighbor distances, and individual admixture.

Given that both environment and space may be influencing population clustering, we then examined the extent to which the environment influences genetic structure while accounting for geographic distance by using the Generalized Dissimilarity Modeling approach (GDM; Ferrier et al. 2007; Fitzpatrick and Keller 2015) in the R package *gdm* (Fitzpatrick et al. 2024) following Mokany et al. (2022). With GDM, we determined if genetic distance was significantly associated with environmental changes and not geographic distance alone. We tested the effects of geographic and environmental distances on genetic distances using the same uncorrelated environmental variables described above for the SuperSOM method. We calculated these effects as linear geographic distances and uncorrected genetic distances in *adegenet* in R. We then estimated pairwise distances among samples for each variable and assessed if environmental distances along with geographic distance significantly predict genetic distance. We also calculated the importance of each variable in the GDM model.

### Timing and mode of divergence

To understand historical demography, which includes the timing of lineage divergence, population size changes through time, and when and if migration occurred, we used the program GADMA (Noskova et al. 2020, 2022). This program uses a genetic algorithm approach to select the best model that jointly infers population size change with time and migration without pre-specifying a model unlike fastsimcoal2 or PipeMaster (Gehara et al. 2020; Excoffier et al. 2021). Therefore, the method assesses various processes such as pure isolation (no migration), isolation with migration, isolation and constant migration, and isolation with secondary contact. GADMA simulates the site frequency spectrum (SFS) using *∂a∂i* or *moments* (Gutenkunst et al. 2009; Jouganous et al. 2017) first based on a random model and then estimates the log likelihood and AICc of this model. It generates new models that are altered randomly or via mutation (randomly changing parameters) or crossover (combining parameters and values from two demographic models). The best of these new models are then selected based on the model with the highest log likelihood and lowest AICc. The final and best model is chosen when log likelihoods cease to change after multiple iterations. We estimated demographic processes between the FL and East lineages using a phylogenetically consistent model where we also included Central and Western lineages given that the former two lineages may not be sister-taxa (see Myers et al. 2024). The inclusion of these additional lineages is also important because parameter estimates from demographic analyses can be biased where migration from unsampled lineages is present (Beerli 2004). We first converted the SNP data to the folded SFS using easySFS (https://github.com/isaacovercast/easySFS) downsampling FL (*n* = 9), East (*n* = 16) and Central-West (*n* = 11) to reduce the sparseness of the SFS matrix. We ran GADMA repeating the genetic algorithm 100 times across 15 processes to achieve convergence. We determined generation time as two years for reaching sexual maturity (Fitch 1963; Ernst and Ernst 2003) and substitution rate at 1.0 × 10^−9^/substitutions/generation (Myers et al. 2024). Because loci showing selection may bias the outcomes of isolation-migration models (Roux et al. 2016), we also ran GADMA without 11 loci that we found to be under significant selection in the genome cline analyses (see below).

### Genome scans

We examined which loci show selection between the FL and East lineages using the R package *pcadapt* (Luu et al. 2017). The program estimates population structure using PCA and admixture for each individual, which we compare to DAPC and *TESS3r* results. With the number of principal components required to predict population structure (here always K = 2), we generated a Z score by regressing the principal components using the pcadapt function. The Mahalanobis distance of each SNP to the mean was then produced. These squared distances when divided by a genomic inflation factor are chi-square distributed with K degrees of freedom and used to calculate a *P* value. To correct *P* values given a false discovery rate, we used the Benjamini-Hochberg procedure (Benjamini and Hochberg 1995) and sorted those *P* values for significantly selected loci below 0.1. We then summed the number of loci showing significant selection for each lineage pair.

### Spatial cline

We predicted the general region and geographical center of the hybrid zone using the R package *akima* (Akima et al. 2016). We used the admixture proportions previously estimated from the *TESS3r* analyses and geographic localities. This predicted the area of 50% admixture, which should be the center of the cline or hybrid zone. We then calculated the geographic gradient of genomic differences between spatially adjacent lineages by estimating the steepness of these differences to generate the width of the cline. Steeper clines have relatively narrower widths than shallow clines. Steep clines may be the result of selection on hybrids or parental species in an environmental cline. These two-dimensional samples were mapped over space and then reduced to a single dimension as required for spatial clinal analyses in the R package *HZAR* (Derryberry et al. 2014). To generate this single dimension, we took the geographic distance between each sample and the center of the hybrid zone estimated from *akima*. A positive or negative sign was assigned to each distance based on the orientation of each individual being on one side or the other of the center line of the admixture cline.

We then estimated the width and center using admixture proportions for each sample and distances to the spatial cline center by fitting these data to the following five sigmoidal clinal models in HZAR using AICc under the Gaussian cline model: (1) no tails, (2) right tail only, (3) left tail only, (4) mirrored tails, and (5) both tails estimated independently (see Derryberry et al. 2014). We ran the MCMC chains for 5 × 10^6^ generations, thinning every 5 × 10^3^ generations. Stationarity was assumed when the estimated sample sizes (ESS) > 200 as assessed in the R package *CODA* for both width and cline center (Plummer et al. 2006).

We also estimated spatial clines for each locus. We calculated spatial cline width and center using HZAR given individual allele frequency. The same procedure using individual distance and orientation to the admixture center line was used to run models. For each locus we also estimated fixation where each allele occurs in >80% of each cline tail (5% of the samples) representing parental lineages. We ran chains for 100,000 generations and these were thinned by 50,000 generations. Finally, for each locus we estimated both spatial cline width and center.

### Genome cline

It has been demonstrated that hybrid zones can change over large geographic distances. For example, such hybrid zones may best be represented as a mosaic with interspersed parental lineages resulting from complex historical changes in the landscape. We therefore used genome clines (Szymura and Barton 1986; Gompert and Buerkle 2011) that estimates selection (or drift) by assessing allelic introgression over admixed individuals regardless of space. Genome clines therefore allows researchers to identify parameters that affect introgression without assuming that clines must have a particular shape (e.g., the smooth, sigmoidal spatial cline). The R package *gghybrid* (Bailey 2024) was used to estimate steepness of the cline on a graph where the X-axis is the hybrid index ordered from one parental lineage to the other and the Y-axis is locus-specific allele frequencies. Significantly steep genome clines (v > 1) demonstrate selection (or drift) on locus introgression. We calculated the center of the genome cline (u), this shows how far alleles introgress between lineages, assuming that the center of the cline is composed of individuals with ~50% admixture and u = 0. Values of v = 1 and u = 0 indicate no deviation from the genome-wide average in frequency of allele copies originating from one parental lineage.

We estimated the genome-wide hybrid index for parental lineages by selecting individuals that show >80% of the genome originating from one parent or the other from the *TESS3r* ancestry coefficients. The genome-wide hybrid index was estimated with 5000 iterations and a burn-in of 2000 based on recommendations from the author (Bailey pers. comm.) using the function esth(). From this we estimated significantly steep loci (v > 1) and the center (u) for each locus given the genome-wide average. Results from three runs were compared to confirm that parameter estimates had converged on similar values. Additionally, to better determine which loci have slopes that are significantly greater than 1, we compared models using wAIC values < −2 where v and u were estimated unconstrained (as above) compared with those where v was fixed at 1.0.

### Locus-environmental interaction

We determined if specific loci were under selection given changes in environment from the FL to East lineages. We used a redundancy analysis (RDA; Forester et al. 2018) to estimate how loci covary relative to a multivariate assessment of environmental data (Rellstab et al. 2015) described for the SuperSOM method above; these represent uncorrelated bioclim, woody plant coverage, elevation, and NDVI for each month. Missing allelic data were imputed from the average value for each locus. The rda function in *vegan* (Dixon 2003) was used to make multiple regressions of the genetic and environmental data yielding a matrix of fitted values. These values were used to make canonical axes of linear combinations of the environmental variables. We calculated the r^2^ value for the model which shows how much variance the environmental data predicts. We used the anova.cca function in *vegan* to estimate the significance of the model. To determine which SNPs were adapted to environmental variables we used SNP loadings from our RDA model and kept those loci occupying 2.5 standard deviations in the tails of the distribution of SNPs (P < 0.015). We removed SNPs that were duplicated across more than one RDA axis. Of those in the final set of SNPs, we determined which environmental variable was most strongly correlated with those significant loci.

### Overlap among genetic metrics

We used Venn diagrams to see the overlap of loci that were significant for: (1) delineating lineages using DAPC, (2) strong spatial clines, (3) genome clines, (4) genome scans, and (5) environmental interactions from RDA. We assessed lineage structure over space and Fst using this subset of loci overlapping among these metrics. We suggest that this reduced-number of loci that retain spatial-lineage structure similar to the entire SNP dataset but with higher Fst values, strongly indicate that selection against introgression is enhancing the boundaries between the East and FL lineages. We used BLAST (Altschul et al. 1990) to identify the loci that were significant among these five metrics and mapped those and all other SQCL loci to the *Ahaetulla prasina* genome (ASM2864084v1; Tang et al. 2023). This taxon represents the closest relative to *Coluber constrictor* (sharing a common ancestor at ~32 my; Burbrink et al. 2020) with an annotated genome sequenced to chromosome level.

## Results

### Lineages and environment

**Fig. 1:**
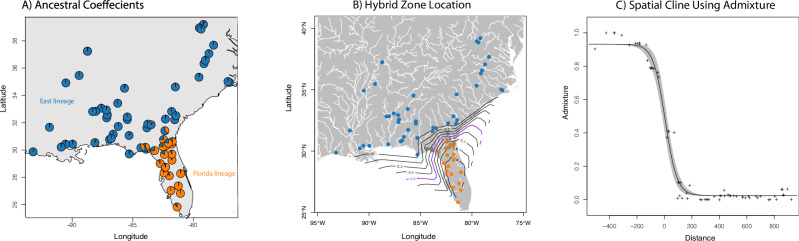
Spatial admixture results for the two lineages of *Coluber constrictor* in the eastern United States. **A** Ancestral coefficients over space using TESS3r. **B** Interpolation of admixture across space showing the location of the hybrid zone. **C** Spatial cline between the two lineages using admixture estimates.

**Fig. 2:**
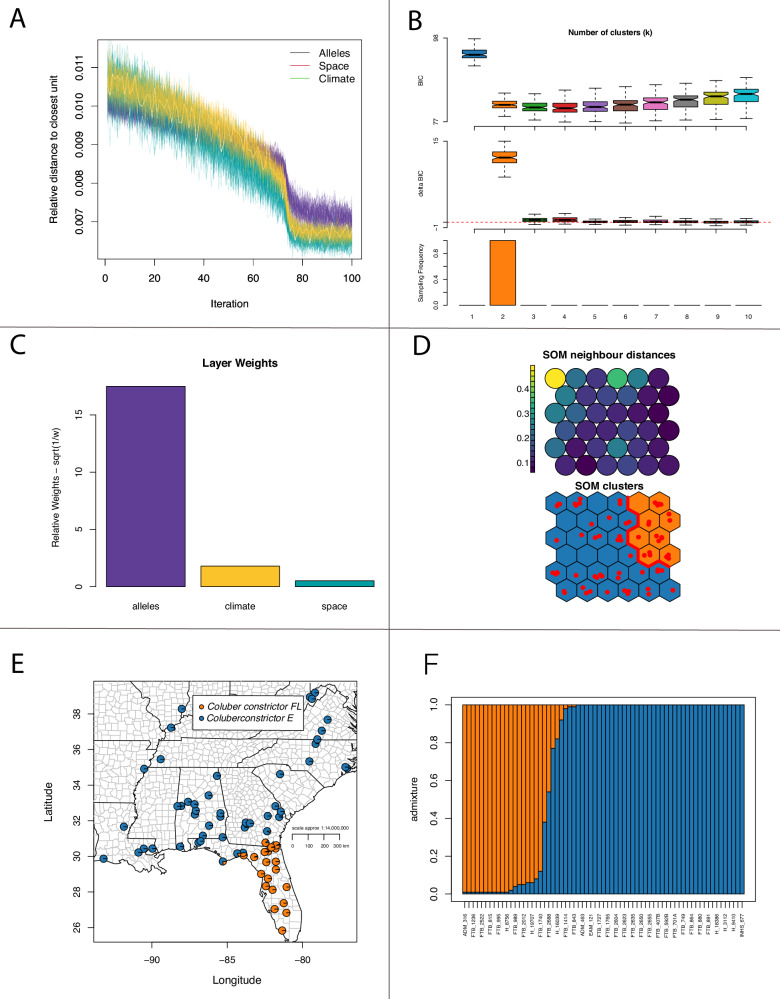
Estimates of groups using self organizing maps (SOM) machine learning methods. **A** Relative distance to the closest unit over each iteration. **B** Support for number of clusters. **C** Data layer weights. **D** SOM neighbor distances and clusters. **E** Map showing the location of individuals and admixture. **F** admixture estimates across all individuals.

With 3143 loci for 67 samples, we found similar results to Myers et al. (2024) for *Coluber constrictor*, but here we focus on two lineages distributed in the Florida peninsula (FL) and the eastern US (East) with a narrow zone of admixture (Fig. 1a). These lineages were also inferred using SOM methods, with admixture estimates significantly correlated between TESS3r and SOM (ρ = 0.984; P = 2.2 × 10^−16^). Importantly, SOM shows that the allelic dataset is driving those clusters separate from environment and space (Fig. 2). These two lineages show an overall pairwise Fst of 0.20.

Using RDA and PCNM and testing significance with CCA ANOVA, we found for the combined samples that the first three spatial structures, which accounted for the broadest neighborhood structures, were significant (*F* = 0.16–0.48; *P* = 0.01–0.03). For the Florida lineage alone, the genetic data were only marginally significant for only the first neighborhood structure (*F* = 0.48, *P* = 0.04). And for the eastern group, with a much larger range, the data were significant for the first three neighborhoods (*F* = 0.019–0.2; *P* = 0.001–0.002).

**Fig. 3:**
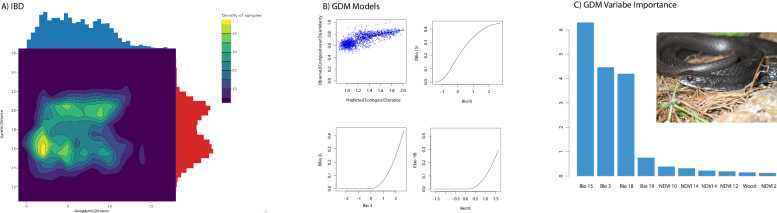
Geographic and environmental association with genetic data. **A** Genetic distance by spatial distance highlighting the presence of two groups. **B** GDM spline model for significant variables. **C** GDM variable importance over all climate variables with inset showing a photo of *Coluber constrictor* from the East lineage taken by F. T. Burbrink.

To further examine the effect of environment and space on genetic structure, we used GDM. Here we show that the environment from subtropical FL to the temperate continent influences population structure despite changes in geographic distance. The GDM model was significant (*P* = 0.00), and when accounting for space, both Bio 3 (isothermality) and Bio 18 (precipitation of the warmest quarter) were significant, with Bio 15 (precipitation seasonality) also having high variable importance (Fig. 3). Plotting genetic distance against geographic distance also shows two distinct genetic groupings, disassociated from distance (Fig. 3). Taken together, the SOM, GDM, and patterns of genetic-geographic distance indicate that these two lineages show a strong effect of isolation by environment (IBE; Figs. 2 and 3).

### Population demography

**Fig. 4:**
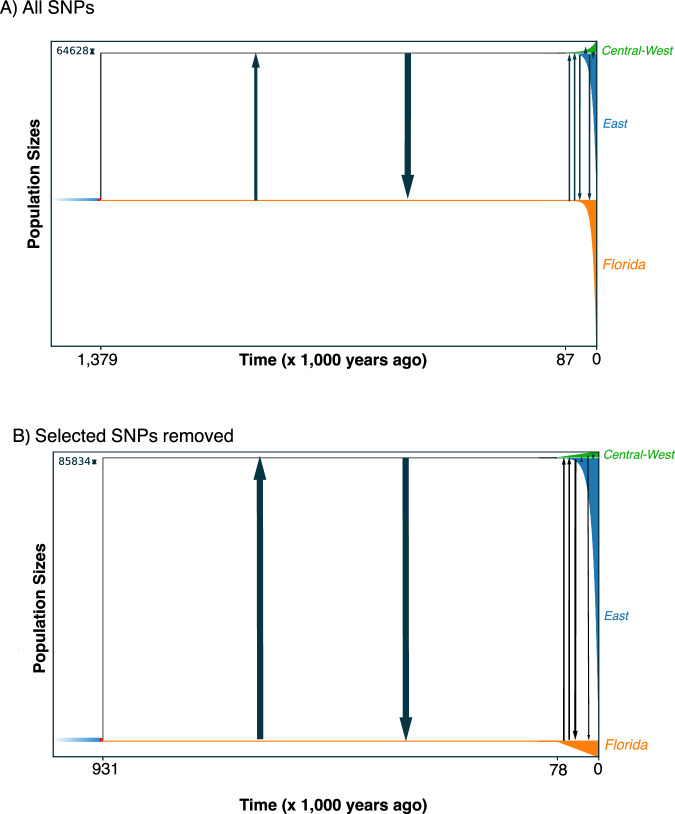
Historical demographic estimates using GADMA. **A** The timing of origin of the Florida, East, and Central-West group, migration rates, and population size changes through time for all SNPs. **B** The timing of origin of the Florida, East, and Central-West group, migration rates, and population size changes through time for SNPs not showing significant selection.

Our three GADMA runs all support a model of isolation and migration (Fig. 4) with the lowest AIC (327.67, ΔAIC compared to the second and third best model = 1.62 and 5.37). These results suggested that the first divergence between Florida and the East/Central-West clade occurred prior to 1.3 mya with divergence between East and the Central-West lineages occurring in the late Pleistocene at ~87 kya. Migration (migrants/generation) among lineages was highest early after lineage divergence, where the East/Central-West lineage received migrants from the FL lineage at a rate of 6.66 and vice versa at 19.98. These rates of migration decreased towards the present day with 0.211 and 2.33 for East receiving FL migrants and FL receiving East, respectively. Given that FL has no current overlap with the Central-West lineage, it is expected that migration is likely non-existence and estimated values of migration ranged from 8.18 × 10^−22^ and 0.022. Migration where East receives Central-West was 0.49 and 1.64 vice versa. When running GADMA without selected loci we found similar divergence estimates, and as expected, slightly higher estimates of migration (Fig. 4). The initial divergence occurred at 0.93 mya with divergence between East and the Central-West lineages at ~78 kya. Migration between lineages was also highest early after lineage divergence where the East/Central-West and FL had the same rates of migration at 19.98. These rates of migration also decreased towards time zero with 0.59 and 3.94 for East receiving FL and FL receiving East, respectively. Migration between FL and the Central-West lineage was low (3.67 × 10^−4^ and 0.33). Finally, migration where East receives Central-West was 0.711 and 1.93 vice versa.

### Hybrid zones and locus characteristics

**Fig. 5:**
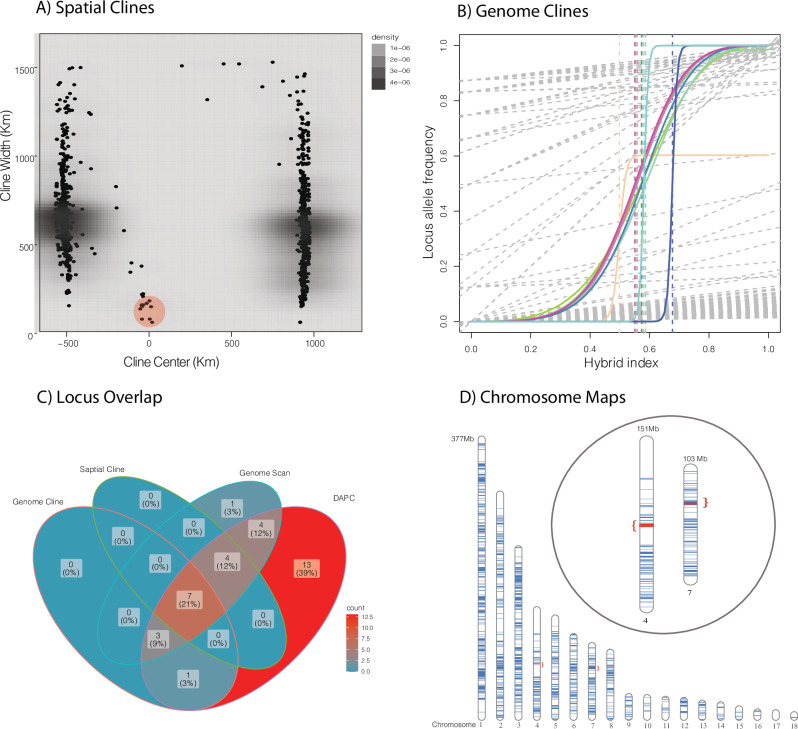
Detecting significant loci isolating the two lineages of *Coluber constrictor*. **A** Spatial cline width vs distance from the cline center (km) and significant loci circled in red. **B** Significant genome clines (color) against background non-significant clines (gray). **C** Venn diagrams showing overlap among significant loci for genome and spatial clines, genome scans, and group prediction via DAPC. **D** SqCL loci from *Coluber constrictor* mapped to the *Ahaetulla prasina* genome (ASM2864084v1) with inset showing the location of significantly selected loci on chromosomes 4 (Z sex chromosome) and 7.

We estimated the location of the center of the hybrid zone using interpolating admixture estimates with geography near the northern border of the Florida peninsula (Fig. 1). Using HZAR with estimates of admixture producing ESS values for all parameters >3000, we predicted a model with no tails and estimated the width of the cline at 167 km (1st to 3rd quartile: 154–180 km) and center of the cline at 5.25 km (1st to 3rd quartile: 0–10 km; Fig. 1). When examining this across all loci, we found spatial cline widths lower than 200 km for 11 loci (Fig. 5). The remaining loci found in two clusters away from the cline center are irrelevant for identifying lineages (not sorted by parentals) and represent SNPs found in only one or a few individuals (Fig. 5a). Examining this for genome clines we discovered 11 loci with a significant slope (median = 3.0 and min/max = 2.39–35.24; Fig. 5). We also found that these same loci, plus one extra locus, were supported as deviating from a fixed model with a slope (v) of 1.0 (wAIC < −2) when using genome cline analyses.

Additionally, using RDA we found 201 loci correlated with environmental data (Bio 3, 5, 8, 12, 15, 17, 18, 19, NDVI 3, 4, 7, 9, 10, and woody plant coverage), dominated by Bio 15 (40 loci), Bio 3 (48 loci), NDVI 10 (47 loci), NDBI 7 (17 loci). Genome scans methods detected 19 significant loci when compared to background loci after adjusting *p*-values (ranging from 1.97 × 10^−21^ to 9.49 × 10^−2^) using the method in Benjamini and Hochberg (1995). We found seven significant loci overlapping among spatial and genome clines, genome scans, and DAPC loci (those loci that define population structure), representing 21% of the pool of significant loci. Three of these loci appear on chromosome 4 (which is likely the Z sex chromosome in snakes), separated by a minimum of 1.48 Mbps and maximum of 23.99 Mbp, and the other four loci appear on chromosome 7, separated by a minimum of 467 Kbp and maximum of 1.98 Mbp (Fig. 5). These all represent protein coding genes from the anchored hybrid enrichment dataset (AHE) or the ultraconserved elements dataset (UCE) with those on Chromosome 4 beingf golgin A4 (AHE), DNA-binding protein SATB1 isoform X1 and X2 (UCE), and phosphodiesterase 1 C (UCE) and those on chromosome 7 being the forkhead box protein P2 (UCE), transcription factor EC and small ribosomal subunit protein eS24-like (UCE), forkhead boxprotein p2 and Protein phosphate 1 regulatory subunit 3a isoform x1 (UCE), and the small ribosomal subunit protein eS24-like MyoD (UCE) family inhibitor domain (Fig. 5). Additionally, these loci are all significantly correlated with precipitation seasonality (Bio 15).

## Discussion

We demonstrate the existence of two deeply divergent lineages of *Coluber constrictor* meeting in a relatively narrow hybrid zone that interfaces the southeastern coastal plains ecoregion of Florida and the southeastern plains of Georgia (Fig. 1). This area is a biodiversity hotspot for many unrelated taxa (Noss et al. 2015). Genetic structure between these lineages is heavily influenced by current climate and adaptation to the subtropical habitats of the Florida peninsula and the temperate habitats of the continental US. These adaptations have likely prevented these two lineages from collapsing via hybridization (Fig. 3). We have discovered seven loci showing strongly resistant alleles to traversing the hybrid zone from one lineage to the other given overlap among significant metrics using genome scans, genome clines, and RDA methods. Given that these loci and others show strong selection with changes in climate, we suggest that these boundaries were likely maintained by strong selection over thousands of generations.

Divergence at the Florida-Continental boundary has been described for many species of vertebrates, insects, and plants (James 1961; Avise 2000; Soltis et al. 2006; Tollis et al. 2012; Devitt et al. 2023; Jones et al. 2023). This divergence in North American racers has produced discrete lineage structure, likely due to isolation by environment and not spurious population clustering as a result of isolation by distance (Fig. 3). Our results present a strong role of the environment for structuring these lineages when considering the following results: estimates of IBD and SOM showing the existence of two groups, GDM showing a significant environment and genetic relationship when accounting for space, and loci showing selection from the environment identified with RDA that are correlated with loci also showing significant slopes with genomes clines through ordered estimates of admixture and spatial clines. Of course, potential selection against hybrids in the hybrid zone may have other causes unrelated to these environments. For racers, the timing of divergence before the mid-Pleistocene indicates that the FL lineage arose within the interglacial cycles that may have isolated parts of central Florida from the remaining continent due to elevated sea levels (Muhs et al. 2003). This would then suggest that the hybrid zone formed when the two lineages met in secondary contact. However, our best-supported model using GADMA suggests that these lineages may never have been completely isolated given the signal of post divergence migration (Fig. 4). This apparent lack of isolation along with our results showing genomic adaptation to unique climates might indicate that ecological speciation was responsible for the formation of these lineages. Alternatively, these lineages may have briefly been isolated and then reconnected rapidly after divergence, though that would be difficult to test with these data. Regarding our results from analyses that excluded selected loci, we found slightly younger estimates of divergence and slightly higher estimates of migration. This also indicated that migration has decreased from the mid-Pleistocene to the Holocene. Therefore, adaptation to these different climate regimes over time likely reduced gene flow as these lineages diverged through the Holocene. Additionally, with decreasing migration, there appears to be massively increasing population sizes in the late Pleistocene for both lineages from GADMA, either including or excluding selected loci. Population expansion in the Pleistocene is common for snakes in North America likely following the retreat of glaciers and amelioration of habitat (Burbrink et al. 2016, 2021, 2022). However, expansion for lineages restricted to the Florida peninsula has only been examined in a few squamates (Fontanella et al. 2008; Guiher and Burbrink 2008; Manthey et al. 2016).

This model of lineage divergence contrasts a previously proposed demographic history of *C. constrictor* (Myers et al. 2024). In that paper, we proposed that this taxon had recently diversified in the absence of gene flow. We suggest that the cause of this may be that GADMA searches all possible demographic models, whereas in our previous analysis we restricted model space to six hypothesized models. If the true demographic model is not included in these kinds of analyses then the outcomes may be biased (Carstens et al. 2017). We suggest that the demographic model showing migration supported by GADMA is likely a better representation of the evolutionary history of these lineages given the admixture proportions shown by *TESS3r* and SOM analyses and the clinal analyses where these two lineages are in geographic contact. Additionally, historical demographic models assume all loci are evolving neutrally and from our other tests there is evidence of selection on some loci. Here we show that isolation and migration parameters are not substantially affected by including a small number of selected loci, though future studies should consider variation among demographic estimates using datasets that include or exclude loci under selection.

Speciation in many taxa occurs prior to the complete cessation of gene flow (Wu 2001; Nosil 2012; Caro et al. 2013; Wang et al. 2019). In fact gene flow in many species continues well over millions of generations past initial species divergence (Price and Bouvier 2002; Hewitt 2011; Barth et al. 2020; Burbrink et al. 2021; Brownstein et al. 2024). Therefore incomplete reproductive isolation may be a stable evolutionary endpoint, especially where hybrids zones serve to filter adaptive alleles from maladaptive alleles (Martinsen et al. 2001; Servedio and Hermisson 2020). Species divergence and their boundaries are therefore maintained at those loci. The East and FL lineages meet in a hybrid zone in northern Florida that has estimated width of 167 km, which is similar in size to other snake clines (Kindler et al. 2017; Burbrink et al. 2021; Fritz et al. 2023) but relatively small compared to combined range of both lineages (the hybrid zone is ~6% of the combined north-to-south range of ~2600 km). We that found selection across a spatial cline agrees with the significant slope for the genome cline that strongly defines these groups across the same seven loci. These loci are correlated with precipitation seasonality (Bio 15) as determined by RDA. This is also an important variable that is correlated with population structure (Fig. 3). Our results suggest that these loci are strongly selected between these two lineages and are likely maintaining lineage boundaries between 49,500 and 690,000 generations since the origin of these lineages. All of these loci represent protein coding genes (six UCEs and one AHE), with four found on chromosome seven and three on chromosome four (likely the Z sex chromosome). The distance between these loci on a single chromosome ranges from 467 Kbp to 23.9 Mbp and would likely span multiple areas of recombination (linkage disequilibrium decay). However, the rates and location of recombination hotspots evolves rapidly in snakes (Hoge et al. 2024; Schield et al. 2020). It is unknown if these clusters of genes are represent a single focus of selection or an inversion, which of course identifies limitations of this target-capture dataset when compared to other approaches, for example genome assemblies from long-read sequencing (e.g., Mérot et al. 2023). It is therefore unclear if these loci or neighboring genes represent the true sources of selection maintaining these lineage boundaries. Moreover, a whole genome approach might find many more loci under selection distributed throughout the genome. We do note that mtDNA differences between these lineages are extreme (Burbrink et al. 2008; Myers et al. 2024) and may reflect rapid divergence and selective sweeps associated with changes in the nuclear genomes. It is possible here, as in some taxa, that species identities are maintained at selection for a few barrier loci (Knief et al. 2019) showing strong phenotypic differences recognized for prezygotic boundaries, reduced postzygotic viability or reproduction, or environmental-selection gradients from the center of the hybrid zone to parental species (Burbrink and Ruane 2021). To understand specifically how the loci identified here interact with the environment and traits isolating these lineages of racers would require a much broader investigation of the hybrid zone using whole genomes and estimates of fitness.

These two lineages of racers should be considered two distinct species that are adapted to unique environments showing isolation by environment as separate from IBD. With regard to ontology and the evolutionary and general lineage species concept (Simpson 1951; Ghiselin 1974; Hull 1978; Wiley 1981; De Queiroz 2007), these two lineages are each composed of populations with unique ancestral descendant relationships and have remained as distinct ontological individuals at least over 465,500 generations despite gene flow. However, we refrain from naming these lineages in this paper because the remaining four lineages of racers would either require taxonomic changes or would render *C. constrictor* sensu stricto paraphyletic (Burbrink et al. 2008; Myers et al. 2024). One way to resolve this would be to elevate the remaining lineages, though identity of the lineage encompassing the type locality of *Coluber constrictor* in Canada (Linnaeus 1758) is unknown (note that Dunn and Wood 1939 suggest the type is from Philadelphia, Pennsylvania). It is preferable then to obtain a wider range of samples among eastern, western, central, and south Texas lineages (see Myers et al. 2024) to better understand interactions among these groups and clearly define ranges in relationship to previously described subspecific names.

Future studies with an annotated genome of *Coluber constrictor* should examine the origins of gene differentiation and islands of divergence in the context of a recombination landscape using long-read whole genomes. With this information, a better understanding of the traits that permit divergence and maintenance of species boundaries between subtropical and temperate North America can be examined. We also underscore that many species show phylogeographic structure in this region and future research should investigate if these patterns are spatially and temporally congruent, and if divergence is occurring across the same region of the genome in snakes.

### Data archiving

All data and code are archived on figshare: https://figshare.com/s/276343b460bed1c04f8e.
